# Watch‐and‐Wait Approach Following Neoadjuvant Chemo‐Radiotherapy for Locally Advanced Rectal Cancer: A Retrospective Single‐Center Cohort Study

**DOI:** 10.1002/jso.28001

**Published:** 2024-11-14

**Authors:** Georgi Kalev, Sylvia Buettner, Tianzuo Zhan, Ralf‐Dieter Hofheinz, Judit Boda‐Heggemann, Christoph Reissfelder, Steffen Seyfried, Georgi Vassilev, Julia Hardt

**Affiliations:** ^1^ Department of Surgery, University Medical Center Mannheim, Medical Faculty Mannheim Heidelberg University Mannheim Germany; ^2^ Department of Biometry and Statistics, University Medical Center Mannheim, Medical Faculty Mannheim Heidelberg University Mannheim Germany; ^3^ Department of Gastroenterology, University Medical Center Mannheim, Medical Faculty Mannheim Heidelberg University Mannheim Germany; ^4^ Department of Hematology and Oncology, University Medical Center Mannheim, Medical Faculty Mannheim Heidelberg University Mannheim Germany; ^5^ Department of Radiation Oncology, University Medical Center Mannheim, Medical Faculty Mannheim Heidelberg University Mannheim Germany

**Keywords:** neoadjuvant therapy, nonoperative management, rectal cancer, watch and wait

## Abstract

**Background and Objectives:**

The watch‐and‐wait (WW) strategy in patients after complete clinical response (cCR) following chemoradiotherapy for locally advanced rectal cancer (LARC) offers the option of organ preservation. The aim of this study was to assess the oncological outcomes of WW patients treated and followed up in a German referral cancer center.

**Methods:**

In this retrospective study, we analyzed the clinical records of consecutive patients with LARC who underwent neoadjuvant radiotherapy/chemoradiotherapy at our institution between January 2020 and December 2023 and received non‐operative management after cCR.

**Results:**

A total of 30 patients undergoing WW for LARC were included. After a median follow‐up of 17 months (SD = 10 months), local regrowth occurred in four patients (4/30, 13.3%), and one patient (1/30, 3.3%) developed distant metastasis. No predictor for tumor regrowth could be identified based on radiological findings at diagnosis, including cT4 and/or cN2, involvement of the mesorectal fascia, extramural vascular invasion or infiltration of the anal sphincter/levator. All patients with local regrowth were successfully surgically treated (R0 resection).

**Conclusion:**

Nonoperative management for patients with cCR after neoadjuvant therapy for LARC proved to be safe. R0 resection was successfully achieved in all patients who underwent salvage surgery.

**Abbreviations**
APR: abdominoperineal resection
CAPOX: capecitabine and oxaliplatin
cCR: complete clinical response
CRT‐CNCT: chemoradiotherapy followed by consolidation chemotherapy (CRT‐CNCT)
CT‐scan: computed tomography scan
DFS: disease‐free survival
EMVI: extramural venous invasion
EUS: endoscopic ultrasound
FOLFOX: folinic acid, 5‐fluorouracil, oxaliplatin
FU: fluorouracil
INCT‐CRT: Induction chemotherapy followed by chemoradiotherapy (INCT‐CRT)
IQR: interquartile range
LARC: locally advanced rectal cancer
LARS: low‐anterior‐resection‐syndrome
LCRCT: long‐course chemoradiotherapy
LR: local recurrence
mFOLFIRINOX: (modified) folinic acid, 5‐fluorouracil, irinotecan, oxaliplatin
MRF: mesorectal fascia
MRI: magnetic resonance imaging
mrMRF+: MRI‐involved mesorectal fascia
ncCR: nearly complete clinical response
OS: overall survival
pCR: complete pathological response
SAS: statistical analysis system
SCRT: short‐course radiotherapy
TME: total mesorectal excision
TNT: total neoadjuvant therapy
VMAT: volumetric modulated arc therapy
WW: watch‐and‐wait

## 1 Introduction

The multimodal treatment of locally advanced rectal cancer (LARC) including neoadjuvant radiotherapy and chemotherapy with fluorouracil followed by rectal resection with total mesorectal excision (TME) provides excellent local tumor control [1]. As complete clinical (cCR) and pathological (pCR) responses were observed in about 10%–15% of patients undergoing neoadjuvant chemoradiation, first attempts were made to treat selected patients achieving cCR within a non‐surgical approach [2, 3, 4, 5]. In this way, the additional morbidity of pelvic surgery after neoadjuvant treatment is avoided. There is also no risk of anastomotic leakage, which, with an incidence of up to 20% after low and ultra‐low resections, contributes mostly to postoperative morbidity after sphincter‐sparing procedures [6, 7]. Furthermore, doubling urinary, fecal, or sexual dysfunction after radiochemotherapy and surgery are avoided. Urinary dysfunction manifests with urinary incontinence, voiding difficulties or urgency and can occur in over 50% of patients [8, 9]. There are reports showing that up to every second patient, particularly after ileostomy reversal, develops severe bowel dysfunction with major Low‐Anterior‐Resection‐Syndrome‐Score (LARS score) [10, 11]. Likewise, postoperative sexual dysfunction is common and is reported to occur in more than 40% of the patients after rectal resection [8]. However, it should be noted that radiotherapy is an independent risk factor for major LARS and can lead to bowel dysfunction in up to 30% of patients [12, 13].

With the introduction of total neoadjuvant chemoradiotherapy using induction or consolidation 5‐FU and oxaliplatin‐based combination chemotherapy, the number of patients with pCR could be doubled compared to standard chemoradiotherapy with fluorouracil (FU) [14, 15]. Consequently, more patients are eligible for non‐surgical treatment and the watch‐and‐wait (WW) strategy is gaining growing interest. However, it should be considered that up to one‐third of the patients managed with WW may develop tumor regrowth after a cCR during the follow‐up [16, 17, 18]. Although tumor regrowth usually occurs locally in the intestinal wall and salvage surgery can be successfully performed in almost all reported cases, there is still uncertainty regarding the oncological safety of the WW strategy [18, 19].

We evaluated data from patients with cCR who received chemoradiotherapy for LARC at our referral center and were managed with a non‐surgical approach. The rate of local tumor regrowth and the success of salvage surgery, as well as the rate of distant metastasis, and disease‐related mortality were assessed. Furthermore, we aimed to identify risk factors for local regrowth.

## 2 Patients and Methods

### 2.1 Patients

In this retrospective study we evaluated the clinical data of all patients who received neoadjuvant therapy for LARC from January 2020 to December 2023 in a primary university center and were managed non‐operatively after achieving a cCR (no clinical evidence of a residual tumor) and an interdisciplinary tumor board decision. Data was extracted by reviewing digital medical records, including inpatient and outpatient visit history. A cCR, defined in accordance with the Memorial Sloan Kettering regression scheme, was identified at the first or at a subsequent follow‐up visit when initially a significant but not complete tumor reduction (nearly complete clinical response—ncCR) was observed [20].

All patients were > 18 years old and had histologically proven rectal adenocarcinoma up to 12 cm above the anal verge based on rigid or flexible rectoscopy. Patients with distant metastases (UICC stage IV) or those who underwent primary rectal resection without neoadjuvant therapy were not included in the analysis.

Staging of all patients comprised pelvic magnetic resonance imaging (MRI) and computed tomography scan (CT‐scan) of thorax, abdomen and pelvis to stage the disease. Transrectal endoscopic ultrasound (EUS) was additionally performed to assess the local tumor spread more precisely, especially in earlier T‐categories.

The data collected in this study is presented in Table 1.

**Table 1:** Summary of the data collected in the study.

Demographic characteristics of the patients	– Age – Sex
Tumor characteristics	– Distance between the distal tumor margin and the anal verge – Depth of tumor penetration (T category according to UICC TNM classification) – Regional lymph node involvement (N category)
MRI findings	– Tumor relation to the mesorectal fascia (MRF). Distance > 1 mm was recorded as an MRI‐involved MRF (mrMRF+) – Extramural venous invasion (EMVI) – (Lateral) lymph nodes – Infiltration of the external anal sphincter or levator ani muscle
Applied radio‐/chemotherapy	
Oncological outcome	– Local regrowth and salvage surgery – Distant metastasis

### 2.2 Radiotherapy Modalities

– Long‐course chemoradiotherapy (LCRCT)
– LCRCT with a total dose of 50.4–54 Gy in 25–28 fractions, targeting the primary tumor as well as the mesorectal, presacral and internal iliac and, if necessary, external iliac lymph nodes [1, 21, 22, 23]. Concurrently, 5‐FU was either administered as continuous intravenous infusion or using the oral prodrug capecitabine.
– Short‐course radiotherapy (SCRT): 5 × 5 Gy over a maximum of 8 days [14].

For both, planning CTs have been acquired in prone position on a Brilliance Big Bore CT (Philips). Radiotherapy treatment planning with volumetric modulated arc therapy (VMAT) was performed with Monaco V6.1 (Elekta AB, Sweden). The treatment has been delivered on a linear accelerator (Synergy or Versa HD, both Elekta AB) with daily cone‐beam CT‐based 3D image guidance.

### 2.3 Neoadjuvant Chemotherapy Before or After SCRT/Chemoradiotherapy

– FOLFOX: oxaliplatin 85 mg/m^2^ (intravenous), leucovorin 400 mg/m^2^ (intravenous), FU 400 mg/m^2^ (intravenous) bolus and FU 2400 mg/m^2^ over 46–48 h by continuous infusion. Treatment cycles were repeated after 14 days. Treatment was administered for 3–4.5 months.
– Alternative:
– CAPOX: 130 mg/m^2^ intravenous oxaliplatin on day 1 and 2000 mg/m^2^ oral capecitabine on days 1–14. Treatment cycles were repeated after 21 days. Treatment was administered for 3–4.5 months.

### 2.4 Immunotherapy

– Pembrolizumab: intravenous infusion 2 mg per kg body weight every 3 weeks (8 cycles of treatment).

The decision on the type of neoadjuvant therapy (radiation modality and type of chemotherapy) was made in a tumor board, taking into account participation in various ongoing prospektive studies.

### 2.5 Surveillance

Up to 8 weeks after completion of the neoadjuvant therapy, tumor re‐staging was performed using digital rectal examination, endoscopic examination with biopsy, MRI of the pelvis and a CT scan of the chest, abdomen, and pelvis [16].

Subsequently, the patients were surveyed with digital rectal examination and a flexible sigmoidoscopy with biopsy every 3–4 months for 2 years and every 6 months for the next 3 years. Pelvic MRI was performed 3–4 times a year in the first 2 years and then yearly in the following 3 years [16]. To exclude distant metastases, CT scans of the chest, abdomen and pelvis were performed annually [16]. For unclear findings, the time intervals were adjusted individually.

Patients with cCR following neoadjuvant chemoradiotherapy who did not undergo TME were classified as having organ preservation. Patients who underwent local transanal excision after neoadjuvant therapy were also considered to have organ preservation.

### 2.6 Statistical Analysis

The statistical analysis was carried out by the Institute for Medical Statistics, Biomathematics and Information Processing at our university center. The data analysis for this manuscript was generated using SAS (Statistical Analysis System) for Windows, Version 9.4 (SAS Institute, Inc., Cary, North Carolina, USA). The patients’ characteristics were summarized depending on the distribution using median and interquartile range or mean and standard deviation. A logistic regression was performed to assess the impact of the risk factors on the occurrence of local regrowth. A significance level of *p* = 0.05 was set.

### 2.7 Ethics

Approval for this study was obtained from the Ethical Committee at our university center with a waiver of informed consent.

## 3 Results

From January 2021 to December 2023, 139 patients with non‐metastatic rectal cancer were treated curatively at our institution and among them 80 patients (51 men and 29 women) with a mean age of 63 years (standard deviation [SD] = 12.5) received neoadjuvant chemotherapy/radiotherapy for LARC UICC Stage II or III. The WW strategy was applied in 30 WW patients (37.5%) with cCR, the other 50 patients underwent rectal resection with TME. In six of the operated patients, a complete pathological response pCR was found in the histopathological specimen. Overall, cCR and pCR was observed in 45% of patients after neoadjuvant chemoradiotherapy. The demographic and tumor‐specific characteristics of WW patients are shown in Table 2. In the WW group, 19 patients underwent LCRCT (12 patients received 54 Gy and 7 patients received 50.4 Gy). In 14 of these patients, a consolidation TNT with FOLFOX/CAPOX was administered. Ten patients underwent SCRT, with nine patients who also received a consolidation FOLFOX/CAPOX. Immunotherapy with pembrolizumab was administered to one patient with microsatellite‐instability–high LARC. The mean follow‐up period was 21 months (SD = 10 months) from the date of diagnosis and 17 months (SD = 10 months) after completion of chemotherapy/radiotherapy, with June 1, 2024 set as a data cut‐off date. Tumor regrowth was observed in five patients. Four of the patients (13%) had a local regrowth at the site of the primary tumor and one patient (3%) developed pulmonary metastases and died 1 year after systemic progression was diagnosed. Local regrowth occurred within a median of 13 months (interquartile range = 12 months) from the end of the neoadjuvant therapy and was detected in all four patients during the follow‐up endoscopy and confirmed histologically. The pelvic MRI showed tumor progression in two patients; while in the other two, rectal wall thickening remained unchanged in the follow‐up MRI. The clinical data and tumor characteristics of these patients are shown in Table 3. In the logistic regression analysis, no statistically significant association of the following variables with local regrowth was found: T4 (*p* = 1.000), N2 (*p* = 1.000), MRF+ (*p* = 1.000), EMVI (*p* = 1.000), infiltration of the anal sphincter/levator (*p* = 1.000). The modality of neoadjuvant therapy—TNT versus no TNT; SCRT versus LCRCT—also had no statistically significant impact on the incidence of local regrowth (*p* = 0.0954 and *p* = 0.25, respectively).

**Table 2:** Demographic data and tumor characteristics of the WW patients.

Demographic data (*n* = 30)	
Age at diagnosis^a^ (years)	65 (12.5)
Sex (female/male)	10/20
**Tumor characteristics**	
Distance to the anal verge^b^^,^ c (cm)	2.5 (3)
Grading	
G1	4
G2	23
G3	3
cT‐category^d^	
cT1	0
cT2	5
cT3	22
cT4	3
cN+^d^	19
mrMRF+^e^ (patients)	11 (36.7%)
Infiltration of the external anal sphincter or levator ani muscle^d^	12 (40%)
EMVI^f^	5 (16.7%)

^a^ Mean (±standard deviation).

^b^ Median (interquartile range).

^c^ Determined by rigid rectoscopy.

^d^ Determined by pelvic MRI or/und trans‐rectal endoscopic ultrasound.

^e^ Involved mesorectal fascia detected on pelvic MRI.

^f^ Extramural venous invasion detected on pelvic MRI.

**Table 3:** Demographic and clinical data of the patients with tumor regrowth after neoadjuvant therapy.

	Patient no. 1	Patient no. 2	Patient no. 3	Patient no. 4	Patient no. 5
Age at diagnosis (years)	69	59	53	78	74
Sex	Male	Female	Female	Male	Male
cT‐category^a^	3	2	2	3	3
cN2‐category^a^	No	Yes	No	No	No
rpT/rpN‐category^b^	rpT2/rpN0	rpT3/rpN0	rpT2/‐	rpT3/rpN0	
Tumor distance to the anal verge^c^ (cm)	2	2	1.5	0.5	5
Infiltration of the external anal sphincter or levator ani muscle^a^	No	No	Yes	Yes	No
mrMRF+^a^, ^d^	No	yes	No	No	No
EMVI^e^	No	No	No	No	—f
Received radiotherapy	SCRT^h^	LCRCT^i^ (50.4 Gy)	LCRCT^i^ (54 Gy)	SCRT^h^	LCRCT^i^ (54 Gy)
TNT^g^ (FOLFOX/CAPOX)	Yes	no	no	Yes	Yes
Tumor regrowth	Local	Local	Local	Local	Distant (lungs)
Salvage surgery	APR^j^	APR^j^	LR^k^	APR^j^	—
Time of tumor regrowth after completion of chemotherapy/radiotherapy (months)	3	14	12	18	8

^a^ Determined by pelvic MRI or/und trans‐rectal endoscopic ultrasound.

^b^ Determined from the submitted specimen[s] after salvage surgery.

^c^ Determined by rigid rectoscopy.

^d^ Involved mesorectal fascia detected on pelvic MRI.

^e^ Extramural venous invasion detected on pelvic MRI.

^f^ Not assessable on mri without contrast agent.

^g^ (Consolidation) total neoadjuvant therapy.

^h^ Short‐course radiotherapy.

^i^ Long‐course chemoradiotherapy.

^j^ Abdominoperineal resection with total mesorectal excision.

^k^ Local resection.

All patients with local regrowth underwent salvage surgery. Endoscopic full thickness resection was performed in one of the patients, while the other three required abdominoperineal resection (APR) with TME. An R0 resection was successfully achieved in all patients, with a minimal margin clearance of <1 mm for the circumferential resection margin in one case. After salvage surgery, the histologic specimen revealed a pathologic stage identical to the clinical stage in two cases; downstaging and upstaging were identified in one case each. In the pathological reports, the TME had a poor quality in two cases (classified as “incomplete”) and excellent quality in one case (classified as “complete”) [24]. No major postoperative complications occurred after salvage surgery. One of the patients with cCR underwent APR due to enterocutaneous fistula formation between the rectum and perianal skin associated with severe symptoms. No tumor cells were detected in the pathological specimen of this patient. A total of 26 patients (87%), including the patient after transanal local excision, needed no further surgery in the observation period, and 4 patients (13%) received a permanent stoma. Among the entire WW group, only the patient with the distal metastasis died during the follow‐up (mortality rate: 3%).

## 4 Discussion

TNT is a recently developed concept where radiotherapy or radiochemotherapy is combined with a platinum‐based multi‐agent chemotherapy (FOLFOX/CAPOX or mFOLFIRINOX) and administered entirely before surgery for rectal cancer. This neoadjuvant approach not only reduces the risk of distant recurrence, but also leads to a significantly higher rate of cCR and pCR compared to conventional regimens, which have opened up a new perspective on the “watch and wait” strategy [14, 15, 16, 20].

In the past few years, the WW approach has become well established at our institution and in this paper, we report on its application in real‐life practice. We analyzed the data of all consecutive patients who received neoadjuvant therapy for rectal cancer between 01/2021 and 12/2023 (*n* = 80) and were non‐operatively managed after achieving cCR. A cCR was identified in 30 patients (37.5%), all of whom were followed up according to the WW surveillance protocol outlined above. Of these 30 patients, 26 (87%) could be handled without a rectum resection. Comparable results were shown in the OPRA study and several case series [5, 16, 17, 25]. The majority of the WW patients in our study received TNT with consolidation chemotherapy (76.7%) and the predominant radiotherapy modality was LCRCT (63.3%). LR occurred in 4/30 WW patients (13.3%) 3–18 months after completion of neoadjuvant therapy, and distant progression was observed in one patient (see Table 2). Other authors report LR in up to one‐third of non‐surgically treated patients that occurred in the first 12–24 months [16, 19, 25, 26, 27, 28]. Considering these data and the median follow‐up of 17 months in our study, it could be assumed that the LR rate of 13% would be an underestimation. Similar to other investigators, we were able to successfully perform salvage R0‐resection in all patients with a LR and achieve excellent pelvic tumor control [16, 27, 28]. A recent systematic review and meta‐analysis showed that the majority of WW patients with local recurrence underwent salvage surgery with a very high R0 resection rate and found no differences in overall survival and in the rate of distant metastasis between the WW strategy group and the surgical treatment group [29] The 2‐ and 5‐year disease‐free survival (DFS) showed no significant difference after adjustment for R0 salvage surgery [29]. In the OPRA study, tumor regrowth was observed in 40% of patients in the INCT‐CRT group and 27% of patients in the CRT‐CNCT group, and these patients were recommended for TME [16]. However, since similar DFS rates were observed in patients who were recommended TME immediately after restaging and in patients who underwent TME after the tumor regrowth [16], it can be assumed that with the scheduled follow‐up with the option of an adequately timed salvage surgery, the concept of WW may be manageable without significantly increasing the risk to the patients. We performed APR with TME in three cases with LR and one patient underwent transanal local excision. In these patients, the primary tumor was located 2 cm or less from the anal verge, so a permanent stoma would have been most likely unavoidable anyway if surgery had been performed immediately after the neoadjuvant therapy. In fact, in 15 patients (50%) in our WW group, the lower edge of the rectal tumor was located 2 cm or less from the anal verge on rigid rectoscopy, which most probably would have required APR with a conventional treatment strategy. Even if an ultra‐low rectal resection without a permanent stoma could be technically feasible in some of the patients, a very low (coloanal) anastomosis would be carried out with potentially poor bowel function. In such patients with low rectal cancer, the WW strategy may be beneficial in terms of functional outcome. A further finding was the absence of tumor cells in the lymph nodes on all pathology reports (pN0), although staging before neoadjuvant therapy revealed cN+ in all three patients who underwent APR. If technically possible, local excision may be an oncologically reasonable alternative to TME for smaller tumors, especially in multimorbid patients. Unfortunately, sphincter preservation is not possible for very low‐lying tumors directly adjacent to the anal sphincter. We did not observe any major complications after salvage surgery. However, it should be noted that the mesorectum was breached in two of the three patients with TME. In the RAPIDO trial [26], mesorectal breach was more frequent in the experimental group and the authors suggested that the prolonged preoperative chemotherapy may have led to a more fragile or fibrotic mesorectum. Whether the longer interval between radiotherapy and salvage surgery is responsible for the poorer specimen quality due to radiation fibrosis and formation of firm scar tissue is to be further investigated. This is in accordance with our finding that TME quality was considered poor in two cases.

Risk factors at first diagnosis such as T4 and/or N2 category, involved MRF, EMVI, which have proven to have substantial influence on oncological outcome (overall survival, DFS, local recurrence) [30, 31, 32] had no significant impact on the regrowth rate in our study. Thus, it can be assumed that the appropriately selected neoadjuvant therapy had successfully reduced the risk of tumor regrowth in the presence of already known risk factors.

Limitations of this retrospective cohort study include the small number of patients enrolled and the absence of a control group. Patients who had no cCR after neoadjuvant therapy and underwent surgery in the same period should not be included in a control group. These patients responded less well to the chemoradiotherapy compared to the WW patients, probably due to their tumor biology, which would have led to a strong selection bias. However, there is currently limited evidence from large prospective randomized controlled trials, so our results contribute to the evidence on the WW strategy in LARC. Another limitation is the relatively short median follow‐up of 17 months.

## 5 Conclusion

In our patient cohort, the WW strategy after cCR following neoadjuvant therapy for LARC proved to be a safe approach with a high rate of organ preservation. Nevertheless, surgical therapy is still an essential pillar in the treatment of rectal cancer with high demands on surgical expertize in patients with local recurrence, especially since local regrowth is not uncommon. In addition, patients with chemoradiotherapy‐induced tumor destruction, may also require rectal resection. Open Access funding enabled and organized by Projekt DEAL.

## Conflicts of Interest

The authors declare no conflicts of interest.

## Synopsis

Watch‐and‐wait strategy for rectal cancer is still to be validated. In this retrospective study on 30 patients with locally advanced rectal cancer who achieved a complete clinical response after neoadjuvant radiotherapy/chemoradiotherapy, the non‐surgical management proved to be safe. All patients with local regrowth underwent a R0 resection.

## Data Availability

The data that support the findings of this study are available on request from the corresponding author. The data are not publicaly available due to privacy and ethical restrictions.
